# Automatic Extraction of Lung Cancer Staging Information From Computed Tomography Reports: Deep Learning Approach

**DOI:** 10.2196/27955

**Published:** 2021-07-21

**Authors:** Danqing Hu, Huanyao Zhang, Shaolei Li, Yuhong Wang, Nan Wu, Xudong Lu

**Affiliations:** 1 College of Biomedical Engineering and Instrumental Science Zhejiang University Hangzhou China; 2 Key Laboratory for Biomedical Engineering Ministry of Education Hangzhou China; 3 Department of Thoracic Surgery II Peking University Cancer Hospital & Institute Beijing China

**Keywords:** lung cancer, clinical staging, information extraction, named entity recognition, relation classification

## Abstract

**Background:**

Lung cancer is the leading cause of cancer deaths worldwide. Clinical staging of lung cancer plays a crucial role in making treatment decisions and evaluating prognosis. However, in clinical practice, approximately one-half of the clinical stages of lung cancer patients are inconsistent with their pathological stages. As one of the most important diagnostic modalities for staging, chest computed tomography (CT) provides a wealth of information about cancer staging, but the free-text nature of the CT reports obstructs their computerization.

**Objective:**

We aimed to automatically extract the staging-related information from CT reports to support accurate clinical staging of lung cancer.

**Methods:**

In this study, we developed an information extraction (IE) system to extract the staging-related information from CT reports. The system consisted of the following three parts: named entity recognition (NER), relation classification (RC), and postprocessing (PP). We first summarized 22 questions about lung cancer staging based on the TNM staging guideline. Next, three state-of-the-art NER algorithms were implemented to recognize the entities of interest. Next, we designed a novel RC method using the relation sign constraint (RSC) to classify the relations between entities. Finally, a rule-based PP module was established to obtain the formatted answers using the results of NER and RC.

**Results:**

We evaluated the developed IE system on a clinical data set containing 392 chest CT reports collected from the Department of Thoracic Surgery II in the Peking University Cancer Hospital. The experimental results showed that the bidirectional encoder representation from transformers (BERT) model outperformed the iterated dilated convolutional neural networks-conditional random field (ID-CNN-CRF) and bidirectional long short-term memory networks-conditional random field (Bi-LSTM-CRF) for NER tasks with macro-F1 scores of 80.97% and 90.06% under the exact and inexact matching schemes, respectively. For the RC task, the proposed RSC showed better performance than the baseline methods. Further, the BERT-RSC model achieved the best performance with a macro-F1 score of 97.13% and a micro-F1 score of 98.37%. Moreover, the rule-based PP module could correctly obtain the formatted results using the extractions of NER and RC, achieving a macro-F1 score of 94.57% and a micro-F1 score of 96.74% for all the 22 questions.

**Conclusions:**

We conclude that the developed IE system can effectively and accurately extract information about lung cancer staging from CT reports. Experimental results show that the extracted results have significant potential for further use in stage verification and prediction to facilitate accurate clinical staging.

## Introduction

### Background

Lung cancer is a group of diseases involving abnormal cell growth in the lung tissue with the potential to invade adjoining parts of the body and spread to other organs. It is the most commonly diagnosed cancer and the leading cause of cancer deaths worldwide [1], which has been a heavy burden on communities and a critical barrier to increasing life expectancy.

Clinical staging of lung cancer plays a critical role in making treatment decisions making and evaluating prognosis [2]. In current clinical practice, clinicians usually decide the clinical staging of lung cancer. Although various advanced diagnostic modalities with high sensitivity and specificity are used by clinical experts, clinical staging still disagrees with pathological staging in approximately one-half of patients, as reported in earlier studies [3,4]. Incorrect clinical staging of lung cancer may result in suboptimal treatment decisions, possibly leading to poor outcomes [3].

As an indispensable examination technique for lung cancer patients, chest computed tomography (CT) provides a large volume of valuable information about the primary tumor and lymph nodes, which is of paramount importance for clinical staging [2,5]. Besides, the reports record the inferences of radiologists about the findings from the images. Although this useful information in the form of natural language is effective and convenient for communication in medical clinical settings, its free-text nature poses difficulties when summarizing or analyzing this information for secondary purposes such as research and quality improvement. Moreover, manually extracting this information is time-consuming and expensive [6,7].

In this study, we aimed to develop an information extraction (IE) system to automatically extract valuable information from CT reports using natural language processing (NLP) techniques to support accurate clinical staging. We first summarized 22 questions about the diagnosis and staging of lung cancer based on the TNM stage guideline [8]. Subsequently, 14 types of entities and 4 types of relations were defined to represent the related information in the CT reports. Using the annotated reports, the following three state-of-the-art deep learning named entity recognition (NER) models were developed to label the entities: iterated dilated convolutional neural networks (ID-CNN) [9], bidirectional long short-term memory networks (Bi-LSTM) [10], and bidirectional encoder representation from transformers (BERT) [11]. Next, a novel relation classification (RC) approach using the relation sign constraint (RSC) was proposed to determine the relations between entities. Finally, a rule-based postprocessing (PP) module was developed to obtain the formatted results by analyzing the entities and relations extracted by NER and RC. We empirically evaluated our system using a real clinical data set. Experimental results showed that the system could extract entities and relations as well as obtain the answers to the questions correctly. Using these extracted results, we can verify the clinical staging accuracy and further develop staging prediction models to alleviate the problem of inaccurate clinical staging.

### Related Works

IE refers to the task of automatically extracting structured semantics (eg, entities, relations, and events) from unstructured text. Cancer information is often extracted from free-text clinical narratives, such as operation notes, radiology, and pathology reports, using rule-based, machine learning, or hybrid methods, which have been widely investigated [12]. In terms of staging information, most studies have extracted only the clinical or pathological stage statements (eg, Stage I, Stage II, and T3N2) but not detailed phenotypes [13-20]. Besides the stage statements, Savova et al [21] and Ping et al [22] extracted some tumor-related information such as the location and size. However, these extracted phenotypes are considerably limited in their ability to support staging, particularly for lymph nodes. To support diagnosis and staging, Yim et al [23] employed a hybrid method to recognize diverse entities and relations from radiology reports for hepatocellular cancer patients, but without further elaboration on how to exploit the extracted information. Chen et al [24] extracted information from various clinical notes including operation notes and CT reports to calculate the Cancer of Liver Italian Program (CLIP) score for hepatocellular cancer patients; however, they provide limited details about the radiology corpus extraction. Bozkurt et al first developed an IE pipeline to extract various types of information from mammography reports [25] and then used the extracted features as the inputs for Bayesian networks to predict malignancy of breast cancer [26].

These rule-based and conventional machine learning methods have extracted information about cancer successfully, and some of them have exploited the extracted results to provide further diagnosis and staging decision support. Nevertheless, the development of hand-craft features and usage of external resources like the Unified Machine Language System (UMLS) and Systematized Nomenclature of Medicine Clinical Terms (SNOMED CT) are time-consuming and can even result in additional propagation errors [10,27,28]. Recently, with the rapid development of deep neural networks, advanced approaches exhibit excellent performance in many NLP tasks without tedious feature engineering [27-33]. Furthermore, some researchers began to adopt these advanced techniques to extract cancer information. Si et al [34] proposed a frame-based NLP method using Bi-LSTM-conditional random field (Bi-LSTM-CRF) to extract cancer-related information by a two-stage strategy. They first identified the keywords in the sentences to determine their frames and then employed models to label the entities in this frame. Using this strategy, they grouped the related entities by different frames. A limitation of this study is that they only evaluated each process in the pipeline using gold standard annotations separately but did not report the overall results of the pipeline. Gao et al [35] proposed a novel hierarchical attention network to predict the primary sites and histological grades of tumors in a text classification manner. Although this approach can directly provide the classification results and show the importance of each word in the text, the scope of the information extracted is considerably limited and insufficient to support cancer diagnosis and staging.

In this study, we aimed to develop an IE system using deep learning methods to extract information about lung cancer staging from CT reports to better support the accurate clinical staging of lung cancer. Our specific contributions involve (1) defining a group of entity types and relation types to cover a wealth of information about lung cancer staging in CT reports, (2) applying advanced deep learning algorithms to develop the IE system, and (3) evaluating the performance of the IE system in a pipeline manner using real clinical CT reports.

## Methods

**Figure 1:**
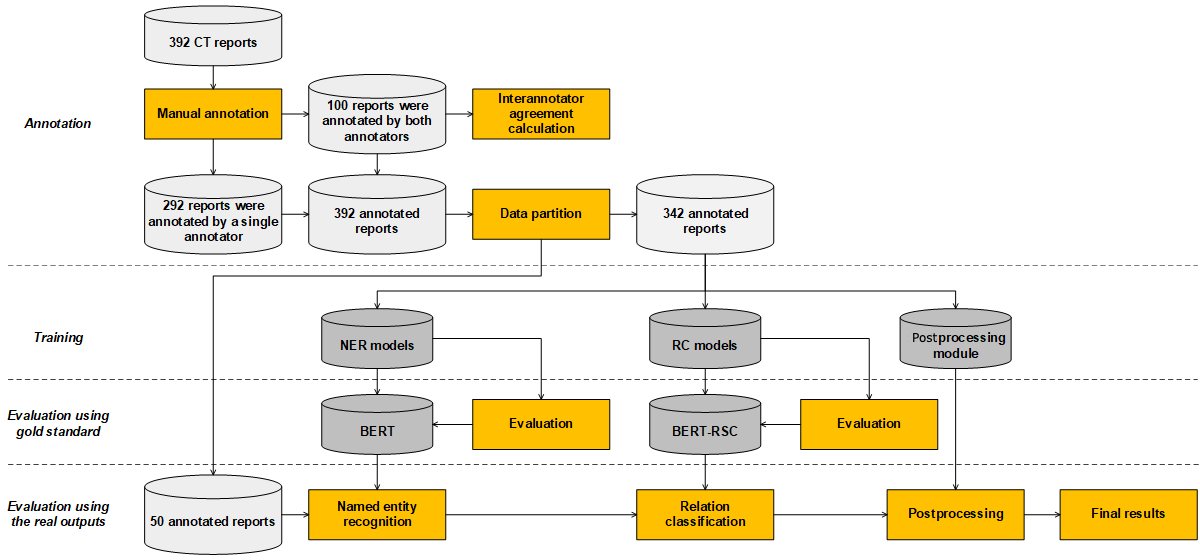
Development process of the information extraction system. BERT: bidirectional encoder representation from transformers; BERT-RC: bidirectional encoder representation from transformers-relation classification; CT: computed tomography; NER: named entity recognition; RC: relation classification.

Figure 1 illustrates the development process of the IE system. First, we annotated the entities and relations in the collected CT reports as the gold standard. Next, the annotated CT reports were used to develop and evaluate the three core parts of the IE system. We also used 50 CT reports to verify the overall performance of the IE system in a pipeline manner. The details of each part are elaborated as follows.

### Data Annotation

In clinical practice, clinicians usually follow the TNM staging guideline to stage the patients. Therefore, we first analyzed the eighth edition of the lung cancer TNM staging summary and parsed it into 41 questions to determine the scope of staging information (Multimedia Appendix 1). Note that the staging guideline covers three aspects of lung cancer (ie, tumor [T], lymph node [N], and metastases [M]), with detailed criteria. Chest CT can hardly provide all the information related to lung cancer staging. Clinicians also use other diagnostic modalities like positron emission tomography (PET), magnetic resonance imaging (MRI), and pathological biopsy to stage the patients. Thus, based on the content of the CT reports, 19 questions were identified under the clinician’s guidance. Moreover, we also included 3 questions about the shape, density, and enhancement extent of the tumors. These 3 questions can facilitate the diagnosis of benign and malignant tumors. All 22 questions are listed in Table 1.

**Figure 2:**
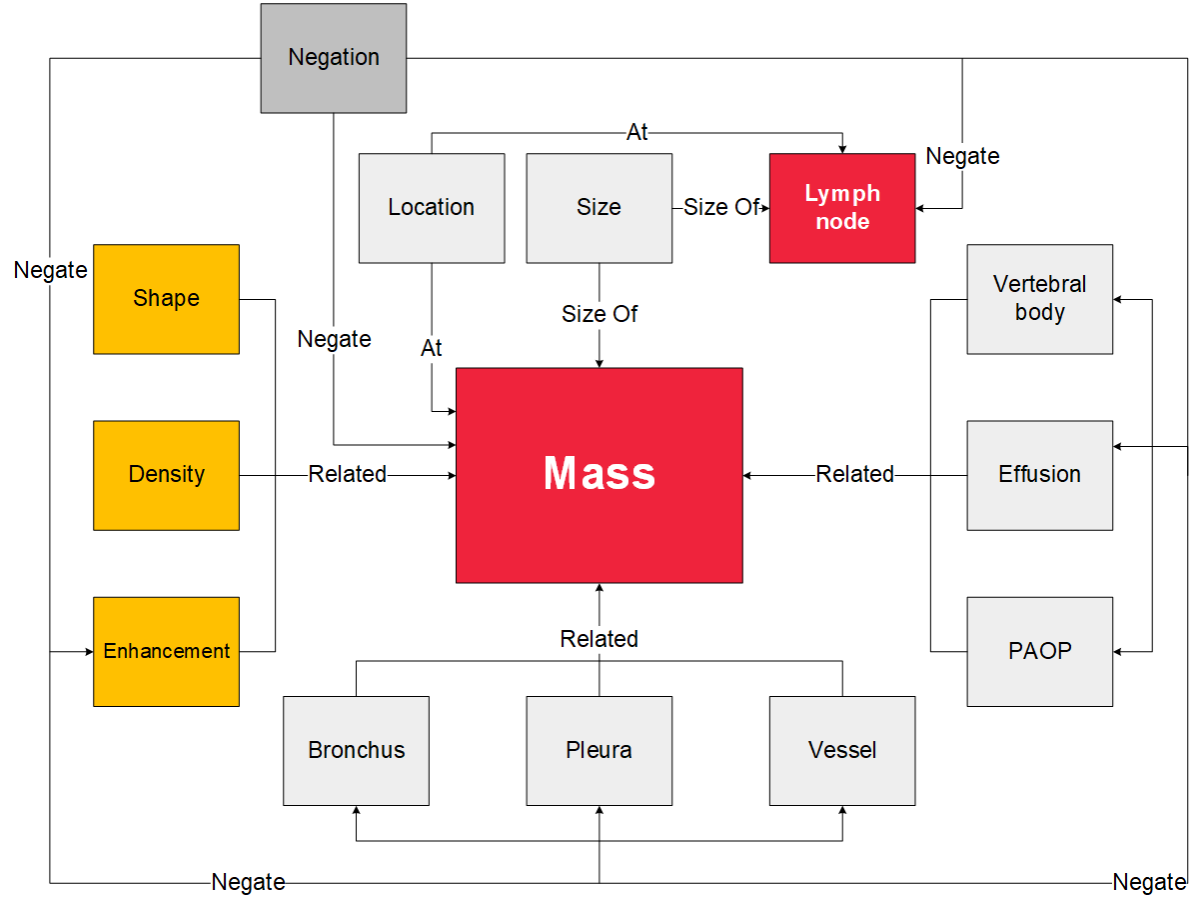
Entity–entity relation map for extracting lung cancer staging information. PAOP: pulmonary atelectasis/obstructive pneumonitis.

Based on the questions listed in Table 1, we defined 14 types of entities and 4 types of relations to represent the staging-related information in the CT reports. Table 2 shows the defined entities. Figure 2 illustrates the entity–entity relation map.

**Figure 3:**
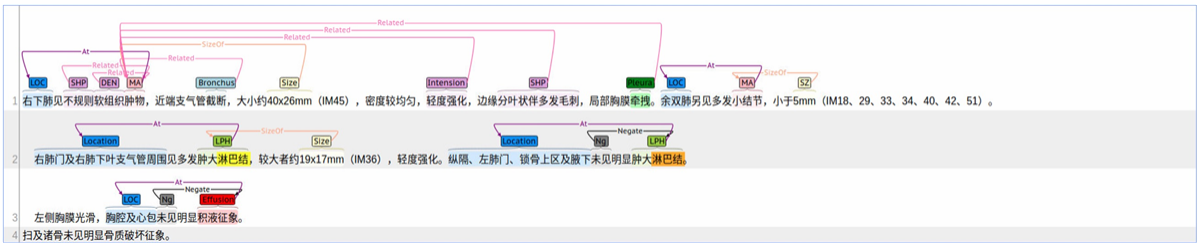
Annotated computed tomography report based on the annotation guideline.

Two medical informatics engineers were recruited to annotate the 392 CT reports by manually following the annotation guideline. The details of the annotation guideline are listed in Multimedia Appendix 2. Note that to obtain the annotation guideline, the annotators first independently annotated 10 reports and discussed the discrepancies until a consensus was reached in consultation with clinicians, resulting in a revised annotation guideline. Using the revised guideline, the annotators independently annotated 10 new reports and repeated the above process. In this manner, the guideline was refined by at least five iterations of annotation, discussion, consultation, and amendment, and then finalized. According to the final annotation guideline, we randomly selected 100 reports for annotation by both annotators to measure the interannotator agreement using the kappa statistic [36]. The remaining 292 reports were annotated only by either of the annotators. The BIO labeling scheme was employed to annotate the data. We employed brat [37] as the annotation tool. Figure 3 shows an example of the annotated CT reports.

**Table 1:** Questions about lung cancer diagnosis and staging^a^.

No.	Question	Type of answer	Stage
1	Whether the tumor can be visualized by imaging or bronchoscopy?	Yes/No	TX
2	What is the greatest dimension of the tumor?	Numerical	T1-4
3	Whether the tumor invades the lobar bronchus?	Yes/No	T1
4	Whether the tumor invades the visceral pleura?	Yes/No	T2
5	Whether there is an atelectasis or obstructive pneumonitis that extends to the hilar region, either involving part of the lung or the entire lung?	Yes/No	T2
6	Whether there is (are) associated separate tumor nodule (s) in the same lobe as the primary?	Yes/No	T3
7	Whether the tumor invades the great vessels?	Yes/No	T4
8	Whether the tumor invades the vertebral body?	Yes/No	T4
9	Whether there is (are) separate tumor nodule (s) in a different ipsilateral lobe to that of the primary?	Yes/No	T4
10	Whether there is regional lymph node metastasis?	Yes/No	N0
11	Whether there is metastasis in ipsilateral hilar lymph nodes, including involvement by direct extension?	Yes/No	N1
12	Whether there is metastasis in ipsilateral mediastinal lymph nodes?	Yes/No	N2
13	Whether there is metastasis in subcarinal lymph nodes?	Yes/No	N2
14	Whether there is metastasis in contralateral mediastinal lymph nodes?	Yes/No	N3
15	Whether there is metastasis in contralateral hilar lymph nodes?	Yes/No	N3
16	Whether there is metastasis in supraclavicular lymph nodes?	Yes/No	N3
17	Whether there is (are) separate tumor nodule (s) in a contralateral lobe?	Yes/No	M1a
18	Whether the tumor with pleural nodules?	Yes/No	M1a
19	Whether there is malignant pleural or pericardial effusion?	Yes/No	M1a
20^b^	What is the shape of the tumor?	Text	NA
21^b^	What is the density of the tumor?	Text	NA
22^b^	What is the enhancement extent of the tumor?	Text	NA

^a^The stages are based on the eighth edition of the lung cancer TNM staging summary.

^b^The questions are not used for staging but are important for diagnosis of benign and malignant tumors.

**Table 2:** Types of entities with descriptions and instances.

Entity type	Description	Instance
Mass	Suspected mass/nodule/lesion in the lung	肿物 (mass)
Lymph node	Suspected lymph node metastasis	肿大淋巴结 (enlarged lymph node)
Location	Location of mass or lymph node	左上肺右基底段 (right basal segment of the upper left lung)
Size	Size of mass or lymph node	25×22 cm
Negation	Negative words	未见 (unseen)
Density	Density of mass	磨玻璃密度 (ground glass density)
Enhancement	Enhancement extent of mass	强化明显 (significant enhancement)
Shape	Shape of mass	边缘见毛刺 (spiculate boundary)
Bronchus	Description of bronchial invasion	支气管狭窄 (bronchial stenosis)
Pleura	Description of pleural invasion or metastasis	胸膜凹陷 (pleural indentation)
Vessel	Description of great vessel invasion	包绕左肺动脉 (surrounds the right lower pulmonary artery)
Vertebral body	Description of vertebral body invasion	椎体见骨质破坏 (bone destruction seen in the vertebral body)
Effusion	Description of pleural or pericardial effusion	心包积液 (pericardial effusion)
PAOP^a^	Description of pulmonary atelectasis or obstructive pneumonitis	肺组织不张 (atelectasis)

^a^PAOP: pulmonary atelectasis/obstructive pneumonitis.

### Word Embedding

As an unsupervised feature representation technique, word embedding maps the words to vectors of real values to capture the semantic and syntactic information from the corpus. In this study, we adopted the word embedding technique pretrained on the Chinese Wikipedia corpus using word2vec [38] for conventional CNN and recurrent neural network (RNN) models. Note that unlike English, Chinese words can be composed of multiple characters but with no space appearing between words. To incorporate the word segmentation information into the NER task, we first used jieba [39], a well-known Chinese text segmentation toolkit, to segment the sentence. Then, we used the randomly initialized real-value vectors to represent whether a character is the first, middle, or last character of the segmented word as in the segmentation embedding. For BERT, we used the default vocabulary to map the tokens to natural numbers.

### NER Process

NER is an essential technique to identify the types and boundaries of the entities of interest, which can drive other NLP tasks [40-43]. Recently developed deep learning NER methods exhibit more powerful performances than the traditional methods without tedious feature engineering [27,29,30,44]. In this study, we selected ID-CNN-CRF, Bi-LSTM-CRF, and BERT to recognize the entities.

ID-CNN is an advanced algorithm extending from the dilated CNN [45]. Instead of simply increasing the depth of a stacked dilated CNN, the ID-CNN applies the same small stack of dilated convolutions multiple times, with each iteration taking the result of the last application as the input to incorporate global information from a whole sentence and alleviate the overfitting problem. Bi-LSTM is another deep learning method using the recurrent neural network architecture that can capture the long-distance dependencies of context from both sides of the sequence and alleviate gradient vanishing or explosion during entity recognition from clinical text. A CRF layer was also employed on the ID-CNN and Bi-LSTM models, as it can exploit the relation constraints among different labels to find the optimal label path for sequence labeling tasks.

BERT is a novel language representation model pretrained on a large corpus using bidirectional transformers [46]. Unlike the traditional embedding methods that can only represent a word with polysemy using one fixed vector, BERT can dynamically adjust the representation depending on the context of the word. It can also be easily fine-tuned to adapt to specific tasks, such as NER, RC, and question answering, and it has shown more powerful performance than conventional CNN and RNN models.

### RC Process

RC is the task of finding semantic relations between pairs of entities, which can group the relevant entities together to generate richer semantics [42,43]. Although traditional RC methods have achieved satisfactory performance [47,48], deep learning RC methods obtained better results and provided an effective way to alleviate the problem of hand-craft features [10,27,28]. In this study, we selected attention-based bidirectional long short-term memory networks (Attention-Bi-LSTM) [32] and BERT to classify the relations between entities.

Note that in this study, two entities in a sentence can only have one type of relation or no relation depending on the definition in Figure 2. For instance, the relation between a lymph node entity and a location entity may be At or NoRelation, but definitely not a SizeOf relation. This information is useful for simplifying the multiclassification problem into a binary classification problem. We propose a novel approach, namely RSC, to use this extra information for RC. Before using the original sentence for relation classification, we first added the tags, namely At, SizeOf, Negate, Related, and NoRelation at the beginning of the sentence (eg, “At<e1>左肺门及纵隔4、5组</e1>见<e2>肿大淋巴结</e2>，较大约14×12m.”). The added At tag is determined based on the two target entity types (location and lymph node). Then, the sentence with the tag can be input into the RC model. Using this method, we can simply incorporate the entity–entity relation constraints into the model to improve the prediction performance.

### PP Step

**Figure 4:**
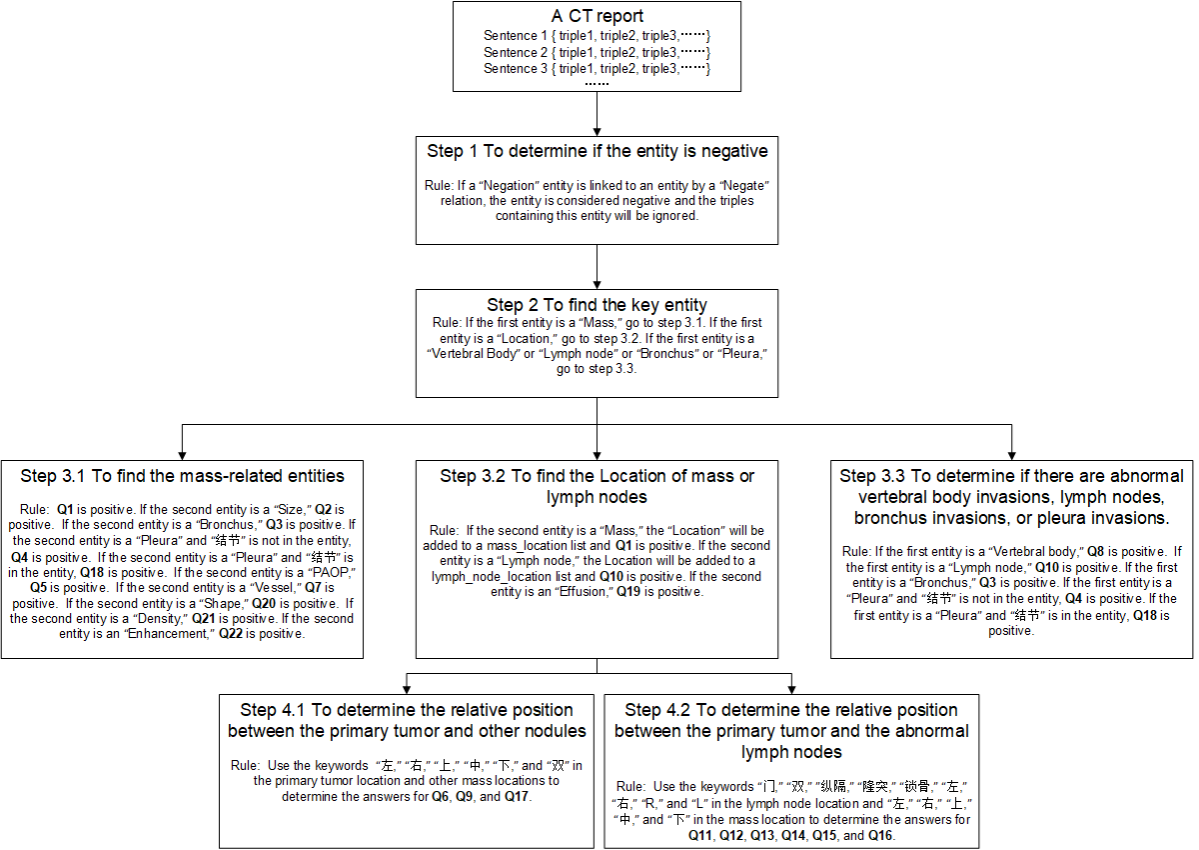
Postprocessing steps.

To obtain the answers to the questions listed in Table 1, it is not enough to directly use the extracted triples (entity 1–relation–entity 2), and further analysis is needed. For example, to answer the question on whether there is metastasis in ipsilateral mediastinal lymph nodes, we first need to know whether there exist a primary tumor and a mediastinal lymph node metastasis for this patient, and then determine the relative position of these two. In this study, we developed a rule-based PP module to process the extracted triples by the NER and RC models. The PP step is presented in Figure 4 and the rules are listed in Multimedia Appendix 3.

### Evaluation Metrics

To evaluate the performance of the models, we used the precision, recall, and F1 score as the evaluation metrics. Moreover, we also employed the microaverages and macroaverages for overall performance evaluation. The corresponding formulations are listed in Multimedia Appendix 4.

## Results

### Data Annotation Results

A total of 392 chest CT reports of lung cancer patients were collected from the Department of Thoracic Surgery II in the Peking University Cancer Hospital. Two medical informatics engineers were recruited to annotate the entities and relations based on the annotation guideline. The statistics of the annotations are summarized in Tables 3 and 4. We had both the engineers annotate 100 CT reports to calculate the interannotator agreement, and the κ values were 0.937 for the entity annotation and 0.946 for the relation annotation, indicating the reliability of the annotation. Prior approval was obtained from the Ethics Committee of the Peking University Cancer Hospital to conduct this study.

**Table 3:** Statistics of annotated named entities.

Entity	Annotated entities, n
Mass	767
Lymph node	492
Location	1748
Size	699
Negation	808
Density	147
Enhancement	146
Shape	437
Bronchus	124
Pleura	262
Vessel	41
Vertebral body	25
Effusion	363
PAOP	78

**Table 4:** Statistics of annotated relations.

Relation	Annotated relations, n
At	1811
SizeOf	683
Related	988
Negate	803

### NER Results

To train and evaluate the NER models, we randomly separated 70% of the CT reports as the training set, 10% as the validation set, and 20% as the test set. The early stopping strategy was used on the validation set to avoid the overfitting problem. The hyperparameters used in this study are listed in Multimedia Appendix 5. We repeated the entire training and evaluation process five times to reduce the possible bias that may be caused by data partitioning.

**Figure 5:**
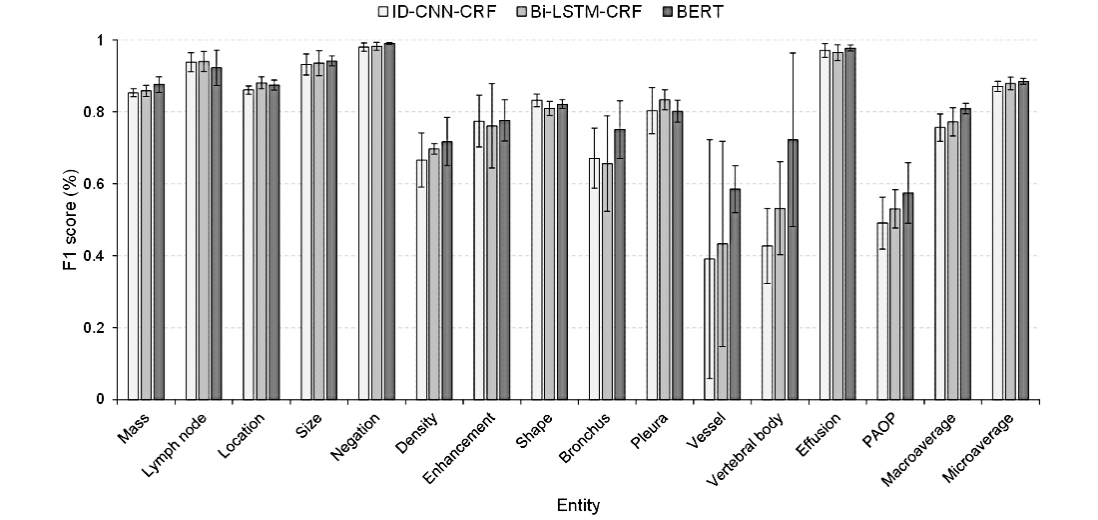
F1 scores with bars showing the standard deviations of the named entity recognition models. Bi-LSTM-CRF: bidirectional long short-term memory networks-conditional random field; BERT: bidirectional encoder representation from transformers; ID-CNN-CRF: iterated dilated convolutional neural networks-conditional random field; PAOP: pulmonary atelectasis/obstructive pneumonitis.

Table 5 and Figure 5 show the results of the NER models. As shown in Table 5, the BERT model achieves the best overall performance with a macro-F1 score of 80.97% and a micro-F1 score of 88.5%. We can notice that the entities with several annotations or plain descriptions (eg, “Lymph Node,” “Negation,” “Size,” and “Effusion”) obtain satisfactory results with F1 scores greater than 90%. However, performances degraded for the entities with a small number of annotations or diverse descriptions (eg, “Shape,” “Pleura,” “Vessel,” “Vertebral Body,” and “PAOP”) Figure 5 shows the results in a more intuitive manner with standard deviations.

**Figure 6:**
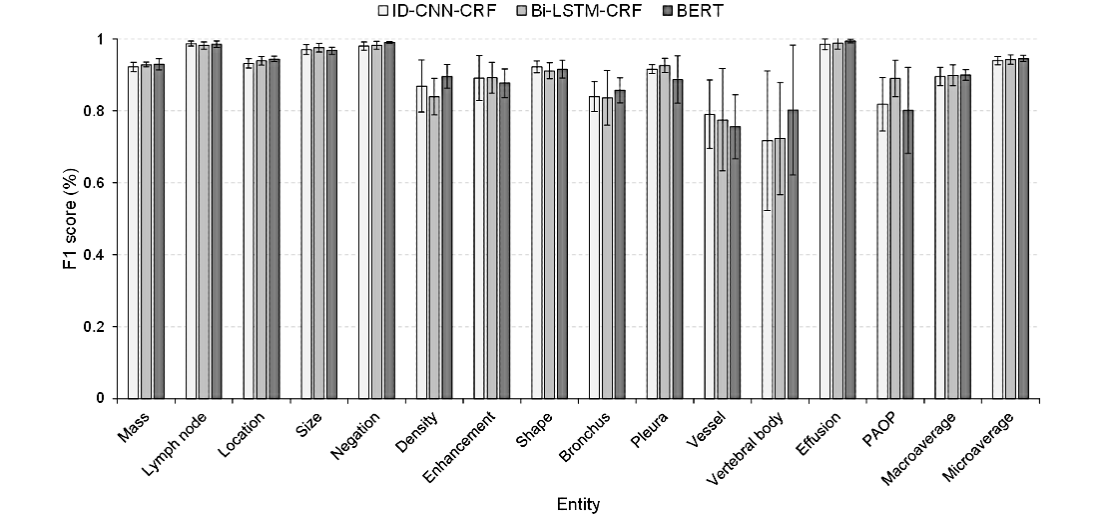
Inexactly matching F1 scores with bar showing the standard deviations of the named entity recognition models. Bi-LSTM-CRF: bidirectional long short-term memory networks-conditional random field; BERT: bidirectional encoder representation from transformers; ID-CNN-CRF: iterated dilated convolutional neural networks-conditional random field; PAOP: pulmonary atelectasis/obstructive pneumonitis.

By further analyzing the extractions, we found that most of the errors were due to an inexact match, where a predicted entity overlapped with the gold standard. For example, the predicted entity “余 (B-Location)肺 (I-Location)内 (O)” is an inexact match for the gold standard annotation “余 (B-Location)肺 (I-Location)内 (I-Location).” Although these extractions could not cover the gold standard exactly, the partially matched entities still contained useful information for RC and PP. We also calculated the inexact matching performances for each type of entity and have presented them in Table 6 and Figure 6.

As shown in Table 6, the macro-F1 scores of ID-CNN-CRF, Bi-LSTM-CRF, and BERT using the inexact metrics are 89.6%, 89.96%, and 90.06%, which obtain improvements of 13.93%, 12.69%, and 9.09% compared with the exact metrics, respectively. Furthermore, the micro-F1 scores of the inexact metrics are all above 94%. Almost all the entities obtain better extraction results under the inexact matching scheme, especially those entities with diverse descriptions, which indicates that the extractions cover most of the annotations.

**Table 5:** Performance of the named entity recognition models.

Entity	ID-CNN-CRF^a^			Bi-LSTM-CRF^b^			BERT^c^		
	Precision (%)	Recall (%)	F1 score (%)	Precision (%)	Recall (%)	F1 score (%)	Precision (%)	Recall (%)	F1 score (%)
Mass	83.11	87.79	85.35	83.86	88.02	85.88	87.92	86.05	87.61
Lymph node	92.29	95.42	93.83	93.29	94.79	94.04	91.52	93.07	92.27
Location	84.85	87.40	86.1	86.99	89.3	88.12	87.93	86.99	87.44
Size	91.6	95	93.24	92.29	94.92	93.56	94.03	94.44	94.22
Negation	97.66	98.45	98.02	97.77	98.79	98.26	99.12	99.11	99.11
Density	64.16	69.66	66.61	68.4	71.47	69.73	75.55	68.49	71.75
Enhancement	74.48	81.04	77.47	74.33	78.4	76.14	81.39	75.03	77.69
Shape	82.65	83.85	83.21	78.95	83.38	81	82.72	81.8	82.2
Bronchus	66.45	67.96	67.11	62.57	69.55	65.66	74.17	76.88	75.1
Pleura	81.48	79.39	80.36	83.54	83.28	83.39	84.59	77.13	80.21
Vessel	37.52	41.59	39.05	44.5	43.13	43.27	68.09	54.53	58.51
Vertebral body	36.43	60.17	42.75	46.52	67.5	53.17	82	66.67	72.24
Effusion	97.02	97.25	97.11	95.77	97.3	96.51	98.32	97.25	97.78
PAOP^d^	47.67	51.33	49.11	50.28	57.25	53.04	65.86	53.2	57.46
Macroaverage	74.1	78.31	75.67	75.65	79.79	77.27	83.8	79.33	80.97
Microaverage	85.85	88.41	87.11	86.56	89.32	87.92	89.28	87.78	88.5

^a^ID-CNN-CRF: iterated dilated convolutional neural networks-conditional random field.

^b^Bi-LSTM-CRF: bidirectional long short-term memory networks- conditional random field.

^c^BERT: bidirectional encoder representation from transformers.

^d^PAOP: pulmonary atelectasis/obstructive pneumonitis.

**Table 6:** Performance of the named entity recognition models calculated using the inexact matching scheme.

Entity	ID-CNN-CRF^a^				Bi-LSTM-CRF^b^				BERT^c^		
	Precision (%)	Recall (%)	F1 score (%)	Precision (%)		Recall (%)	F1 score (%)	Precision (%)		Recall (%)	F1 score (%)
Mass	89.78	94.81	92.19	90.71		95.2	92.89	94.11		92.05	93.02
Lymph node	97.11	100.42	98.73	97.43		99	98.2	97.8		99.41	98.59
Location	91.88	94.66	93.24	92.73		95.2	93.95	95.01		93.99	94.47
Size	95.33	98.9	97.06	96.28		99.06	97.62	96.58		97.03	96.79
Negation	97.66	98.45	98.02	97.77		98.79	98.26	99.12		99.11	99.11
Density	84.09	90.53	86.95	82.39		86.13	84.01	94.48		85.49	89.64
Enhancement	85.64	93.26	89.11	86.79		92.3	89.25	91.53		85.03	87.73
Shape	91.59	92.92	92.22	88.76		93.94	91.16	92.23		91.12	91.6
Bronchus	83.20	85.03	84.01	79.4		89.19	83.73	84.76		87.76	85.75
Pleura	93.07	90.44	91.67	92.86		92.54	92.67	93.12		85.7	88.73
Vessel	81.29	79.18	79.09	84.66		73.82	77.52	89.03		67.5	75.58
Vertebral body	63.81	92.5	71.76	65.76		86.67	72.28	92		73.33	80.24
Effusion	98.4	98.64	98.5	98.18		99.74	98.93	100		98.91	99.45
PAOP^d^	80.1	84.64	81.84	84.23		96.39	89.03	90.23		74.99	80.13
Macroaverage	88.07	92.46	89.6	88.43		92.71	89.96	93.57		87.96	90.06
Microaverage	92.66	95.42	94.01	92.87		95.84	94.32	95.39		93.81	94.57

^a^ID-CNN-CRF: iterated dilated convolutional neural networks-conditional random field.

^b^Bi-LSTM-CRF: bidirectional long short-term memory networks- conditional random field.

^c^BERT: bidirectional encoder representation from transformers.

^d^PAOP: pulmonary atelectasis/obstructive pneumonitis.

### RC Results

To evaluate the proposed RC method, the data set was randomly separated such that 70%, 10%, and 20% of the CT reports were used as the training, validation, and test sets, respectively. Attention-Bi-LSTM and BERT were selected as the baselines. The annotated entities were provided in this step for evaluating the performance of the RC models. The hyperparameters of the RC models are listed in Multimedia Appendix 5. We also repeated the entire training and evaluation process five times with different random seeds to alleviate the possible bias caused by data partitioning.

**Figure 7:**
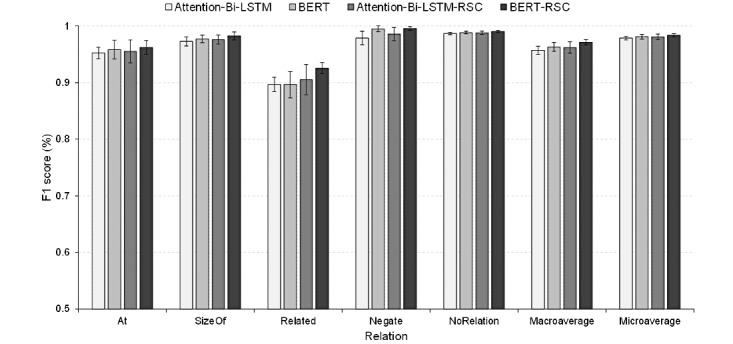
F1 scores with bars showing the standard deviations of the relation classification models. Attention-Bi-LSTM: attention-based bidirectional long short-term memory networks; Attention-Bi-LSTM-RSC: attention-based bidirectional long short-term memory-relation sign constraint; BERT: bidirectional encoder representation from transformers; BERT-RSC: bidirectional encoder representation from transformers-relation sign constraint.

Table 7 and Figure 7 show the experimental results of the RC models. As depicted in Table 7, all the four models achieve excellent performances with macro-F1 values above 95% and micro-F1 values above 97%. Comparing the baseline and proposed methods indicates that the RSC improves the performances of both the baseline models, especially for the Related RC. Moreover, the BERT-RSC achieves the best performance among all the models.

**Table 7:** Performance of the proposed and baseline relation classification models.

Relation	Baseline								Proposed						
	Attention-Bi-LSTM^a^				BERT^b^				Attention-Bi-LSTM-RSC^c^				BERT-RSC^d^		
	Precision (%)	Recall (%)	F1 score (%)	Precision (%)		Recall (%)	F1 score (%)	Precision (%)		Recall (%)	F1 score (%)	Precision (%)		Recall (%)	F1 score (%)
At	96.02	94.47	95.23	96.3		95.39	95.83	96.25		94.79	95.5	96.95		95.55	96.23
SizeOf	97.05	97.51	97.27	98.13		97.35	97.73	97.19		98.1	97.61	98.11		98.42	98.25
Related	88.17	91.47	89.65	85.22		94.7	89.64	88.95		92.31	90.55	89.17		96.27	92.56
Negate	98.7	97.07	97.87	99.38		99.63	99.5	99.33		97.82	98.56	99.38		99.74	99.56
NoRelation	98.7	98.67	98.68	99.11		98.57	98.84	98.83		98.77	98.8	99.22		98.87	99.05
Macroaverage	95.73	95.84	95.74	95.63		97.13	96.31	96.11		96.36	96.2	96.57		97.77	97.13
Microaverage	97.88	97.88	97.88	98.11		98.11	98.11	98.08		98.08	98.08	98.48		98.48	98.37

^a^ID-CNN-CRF: iterated dilated convolutional neural networks-conditional random field.

^b^Bi-LSTM-CRF: bidirectional long short-term memory networks- conditional random field.

^c^BERT: bidirectional encoder representation from transformers.

^d^BERT-RSC: bidirectional encoder representation from transformers-relation sign constraint.

### PP Results

Based on the experimental results presented above, we selected the BERT model for NER and RC. Note that instead of using the annotated data, we directly used the output of the NER model as the input for RC and employed the PP module to analyze the triples extracted by NER and RC to verify the performance of the IE system. We randomly selected 50 reports, for which both the annotators manually answered the 22 questions. Table 8 shows the number of positive answers annotated to each question in the 50 reports and the experimental results of the IE system for each question. The experimental results prove that the IE system achieves a macro-F1 score of 94.57% and a micro-F1 score of 96.74%, indicating that the system can effectively extract information related to lung cancer staging from CT reports.

By analyzing the incorrect answers, we found that the main reason for inaccurate extraction was that some entities or relations were not recognized by the system. For example, missing “Mass” or “At” relations made it impossible to determine the relative position between the primary tumor and other nodules, resulting in low recall values of Q6, Q9, and Q18. Besides, missing “Bronchus,” “PAOP,” “Vessel,” “Density,” and “Enhancement” entities led to low recall values of Q3, Q5, Q7, Q21, and Q22 when relevant descriptions were inherently scarce.

**Table 8:** Experimental results of the developed information extraction system.

No.	Number of positive answers annotated	Precision (%)	Recall (%)	F1 score (%)
1	50	100	100	100
2	47	97.83	95.74	96.77
3	16	100	87.50	93.33
4	27	100	96.3	98.11
5	6	83.33	83.33	83.33
6	17	100	82.35	90.32
7	5	100	80	88.89
8	2	100	100	100
9	14	100	85.71	92.31
10	28	100	100	100
11	18	100	100	100
12	22	95.45	95.45	95.45
13	1	100	100	100
14	19	95	100	97.44
15	6	100	100	100
16	5	100	100	100
17	20	95	95	95
18	5	80	80	80
19	2	100	100	100
20	28	96.3	92.86	94.55
21	14	100	85.71	92.31
22	16	85.71	80	82.76
Macroaverage		96.76	92	94.57
Microaverage		97.49	95.99	96.74

## Discussion

### Principal Findings

In this study, we developed an IE system to extract information related to lung cancer staging from CT reports automatically. The experimental results indicate that the IE system can effectively extract the useful entities and relations using the NER and RC models and accurately obtain the answers to the questions about lung cancer staging using the PP module. The extracted information shows significant potential to support further research about accurate lung cancer clinical staging.

Although the macro-F1 score of NER is only 80.97%, which seems insufficient to support RC and PP, the IE system still achieves satisfactory results. The main reason is that the PP module exploits the key characters in the extracted entities or only the presence of the entities to obtain the answers but does not need the complete entities. For example, the annotation of the sentence “右肺下叶基底段见软组织密度肿块” is [Location_B, Location_I, Location_I, Location_I, Location_I, Location_I, Location_I, O, Mass_B, Mass_I, Mass_I, Mass_I, Mass_I, Mass_I, Mass_I], but the NER result is [Location_B, Location_I, Location_I, Location_I, Location_I, O, Location_I, O, Mass_B, Mass_I, Mass_I, Mass_I, Mass_I, Mass_I, Mass_I], which means the Location entity extracted is merely “右肺下叶基.” However, this partial Location entity is correctly linked to the Mass entity “软组织密度肿块” with an “At” relation by the RC model, and the key characters “右” and “下” in the Location entity can support the following PP step. The high macro-F1 and micro-F1 of the inexact matching scheme indicate that most of the entities can be extracted completely or partially by the NER model. Furthermore, the extractions cover most of the key characters needed during the PP step.

For the RC task, all the four models achieve satisfactory performances. This is because the descriptions are similar in many sentences so that the models can easily learn these patterns. However, for the Related relation, none of the models obtain the perfect performance. The main reason is that some types of entities like “Vertebral Body” and “Vessel” are rare and have diverse descriptions, making it difficult for these models to learn the corresponding patterns. The addition of RSC may make the descriptions more uniform so that the models may learn the patterns more easily.

For the NER and RC tasks, the advanced pretrained BERT model achieves better performance compared to the conventional CNN and RNN methods, thus verifying the superiority of large language representation models for various NLP tasks.

### Limitations

Although the rule-based PP module can accurately obtain the answers to the defined questions by analyzing the extracted entities and relations, these hard-coded rules are difficult to maintain and update. Furthermore, for better use of clinical knowledge (eg, enlarged lymph nodes with a minimum diameter greater than 10 mm are often considered metastatic), we need to establish a more comprehensive knowledge base to analyze the extracted information. Ontology, as a formal representation of medical knowledge, has become the standard method to develop knowledge bases [49,50]. In future, we can use the Web Ontology Language (OWL) [51] to construct the knowledge graph and employ the Semantic Web Rule Language (SWRL) [52] to develop the reasoning rules for lung cancer staging.

In this study, we explored the feasibility of extracting information related to lung cancer staging from CT reports using an NER+RC+PP pipeline in a single hospital. When generalizing this approach to other hospitals, the entity and relation definitions as well as the annotation strategy can be important references for the same application, and the developed pipeline can also be reused. However, if researchers want to customize the entity types or relation types to suit their purpose or if the writing style of CT reports is significantly different from that in the reports that we used, fine-tuning of BERT using the newly annotated reports may be a possible way to obtain satisfactory generalization.

### Future Research

In the current study, pathological staging was not applied as the gold standard to evaluate the correctness of the extracted results. This is mainly because in clinical practice, clinicians use not only CT but also PET, MRI, and other diagnostic modalities to stage patients. Therefore, it is insufficient to use only the information extracted from the CT report to stage the patients. In future, we plan to extract staging information from other examination reports and use this multisource information to verify the staging correctness from a more comprehensive perspective. Moreover, by combining various details such as laboratory tests, disease history, and radiomics data, we can employ advanced machine learning algorithms to develop clinical staging prediction models to further alleviate the large number of disagreements between clinical and pathological stages.

### Conclusions

In this study, we developed an IE system to extract lung cancer staging information from CT reports automatically using NLP techniques. Experimental results obtained using real clinical data demonstrated that the IE system could effectively extract the relevant entities and relations using the NER and RC models. It could also accurately answer the staging questions using the rule-based PP module, thus proving the potential of this system for lung cancer staging verification and clinical staging prediction.

## Appendix

Multimedia Appendix 1 Parsed questions about lung cancer staging.

Multimedia Appendix 2 Annotation guideline.

Multimedia Appendix 3 Postprocessing rules.

Multimedia Appendix 4 Evaluation metrics.

Multimedia Appendix 5 Hyperparameters of the named entity recognition and relation classification models. Attention-Bi-LSTM-RSC: attention-based bidirectional long short-term memory-relation sign constraint; Bi-LSTM-CRF: bidirectional long short-term memory networks-conditional random field; BERT: bidirectional encoder representation from transformers; BERT-RSC: bidirectional encoder representation from transformers-relation sign constraint; ID-CNN-CRF: iterated dilated convolutional neural networks-conditional random field.

## Abbreviations

Attention-Bi-LSTM: attention-based bidirectional long short-term memory networks
Bi-LSTM: bidirectional long short-term memory networks
Bi-LSTM-CRF: bidirectional long short-term memory networks- conditional random field
BERT: bidirectional encoder representation from transformers
CLIP: Cancer of Liver Italian Program
CRF: conditional random field
CT: computed tomography
ID-CNN: iterated dilated convolutional neural networks
ID-CNN-CRF: iterated dilated convolutional neural networks-conditional random field
IE: information extraction
MRI: magnetic resonance imaging
NER: named entity recognition
NLP: natural language processing
OWL: Web Ontology Language
PAOP: pulmonary atelectasis/obstructive pneumonitis
PET: positron emission tomography
PP: postprocessing
RC: relation classification
RNN: recurrent neural network
RSC: relation sign constraint
SNOMED CT: Systematized Nomenclature of Medicine Clinical Terms
SWRL: Semantic Web Rule Language
UMLS: Unified Machine Language System
