# Supplementary material for: Automatic Extraction of Lung Cancer Staging Information From Computed Tomography Reports: Deep Learning Approach
Source: JMIR Med Inform. 2021 Jul 21;9(7):e27955. doi: 10.2196/27955 (PMC8339987; doi:10.2196/27955)
Supplement: Multimedia Appendix 1 [file medinform_v9i7e27955_app1.pdf]

Multimedia Appendix 1. The parsed questions about lung cancer staging.

Stage	Questions	Type of Answer	Whether can be answered based on CT reports
TX	Whether the tumor is proven by the presence of malignant cells in sputum or bronchial washings?	Yes/No	
TX	Whether the tumor can be visualized by imaging or bronchoscopy?	Yes/No	Q1
T0	Whether is carcinoma in situ?	Yes/No	
T1-4	What is the greatest dimension of the tumor?	Numerical (cm)	Q2
T1mi	What is the greatest dimension of the tumor invasion?	Numerical (mm)	
T1mi	Whether the tumor with a predominantly lepidic pattern?	Yes/No	
T1	Whether the tumor invades the lobar bronchus?	Yes/No	Q3
T2	Whether the tumor invades the main bronchus?	Yes/No	
T2	Whether the tumor invades the visceral pleura?	Yes/No	Q4
T2	Whether there is a atelectasis or obstructive pneumonitis that extends to the hilar region, either involving part of the lung or the entire lung?	Yes/No	Q5
T3	Whether the tumor invades the chest wall (including superior sulcus tumors)?	Yes/No	
T3	Whether the tumor invades the phrenic nerve?	Yes/No	
T3	Whether the tumor invades the parietal pericardium?	Yes/No	
T3	Whether there is(are) associated separate tumor nodule(s) in the same lobe as the primary?	Yes/No	Q6
T4	Whether the tumor invades the diaphragm?	Yes/No	
T4	Whether the tumor invades the mediastinum?	Yes/No	
T4	Whether the tumor invades the heart?	Yes/No	
T4	Whether the tumor invades the great vessels?	Yes/No	Q7
T4	Whether the tumor invades the trachea?	Yes/No	
T4	Whether the tumor invades the recurrent laryngeal nerve?	Yes/No	
T4	Whether the tumor invades the esophagus?	Yes/No	
T4	Whether the tumor invades the vertebral body?	Yes/No	Q8
T4	Whether the tumor invades the carina?	Yes/No	
T4	Whether there is(are) separate tumor nodule(s) in a different ipsilateral lobe to that of the primary?	Yes/No	Q9

N0	Whether there is regional lymph node metastasis?	Yes/No	Q10
N1	Whether there is metastasis in ipsilateral peribronchial lymph nodes, including involvement by direct extension?	Yes/No	
N1	Whether there is metastasis in ipsilateral hilar lymph nodes, including involvement by direct extension?	Yes/No	Q11
N1	Whether there is metastasis in intrapulmonary nodes, including involvement by direct extension?	Yes/No	
N2	Whether there is metastasis in ipsilateral mediastinal lymph nodes?	Yes/No	Q12
N2	Whether there is metastasis in subcarinal lymph nodes?	Yes/No	Q13
N3	Whether there is metastasis in contralateral mediastinal lymph nodes?	Yes/No	Q14
N3	Whether there is metastasis in contralateral hilar lymph nodes?	Yes/No	Q15
N3	Whether there is metastasis in ipsilateral or contralateral scalene lymph nodes?	Yes/No	
N3	Whether there is metastasis in supraclavicular lymph nodes?	Yes/No	Q16
M0	Whether there is distant metastasis?	Yes/No	
M1a	Whether there is(are) separate tumor nodule(s) in a contralateral lobe?	Yes/No	Q17
M1a	Whether the tumor with pleural nodules?	Yes/No	Q18
M1a	Whether the tumor with pericardial nodules?	Yes/No	
M1a	Whether there is malignant pleural or pericardial effusion?	Yes/No	Q19
M1b	Whether there is single extrathoracic metastasis in a single organ?	Yes/No	
M1c	Whether there is multiple extrathoracic metastasis in one or several organs?	Multiple metastasis in one organs/ multiple metastasis in several organs/No	
