# Supplementary material for: Automatic Extraction of Lung Cancer Staging Information From Computed Tomography Reports: Deep Learning Approach
Source: JMIR Med Inform. 2021 Jul 21;9(7):e27955. doi: 10.2196/27955 (PMC8339987; doi:10.2196/27955)
Supplement: Multimedia Appendix 2 [file medinform_v9i7e27955_app2.pdf]

Multimedia Appendix 2. Annotation guideline.

1. Annotation principles for each type of entity

1.1 Mass entity

Annotating the words describing the suspected mass, e.g., “肿物/肿块 (mass)”, “结节/结节灶 (nodule)”, “结节影/肿块影/密度影/软组织影 (nodule shadow/mass shadow/density mass/soft tissue shadow)”, and etc., as the “Mass” entity. Note that the descriptions not indicating suspected mass like “远端见肺不张影 (see distal atelectasis shadow)”, “斑片状渗出影 (patchy exudative shadow)” are not annotated.

1.2 Lymph nodes entity

Annotating the words describing the suspected lymph nodes metastasis, e.g., “肿大淋巴结 (swollen lymph nodes)”, “淋巴结增大 (enlarged lymph nodes)”, and etc., as the “Lymph Nodes” entity. Note that the description “钙化淋巴结 (Calcified lymph nodes)” not indicating lymph nodes metastasis are not annotated.

1.3 Location entity

Annotating the consecutive words describing the locations, e.g., “左肺下叶外基底段 (left lower lobe outer basal segment)”, “双侧锁骨上区及纵膈内 (bilateral supraclavicular region and mediastinum)”, and etc., as the “Location” entity. Note that if the location relating to some uninterested description, e.g., “双肺见蜂窝状改变 (see honeycomb changes in both lungs)”, do not annotate.

1.4 Size entity

Annotating the words describing the size of suspected mass and lymph nodes metastasis, e.g., “24×19mm”, “9mm”, “＜5mm”, and etc., as the “Size” entity. Note that we only annotated the value, symbols, and units as the Size entity, but not including the words like “约 (approx.)”, “大小约

(size approx.)”.

1.5 Negation entity

Annotating the words indicating negative, e.g., “未见 (no)”, as the “Negation” entity.

1.6 Density entity

Annotating the words describing the density of the suspected mass, e.g., “磨玻璃密度 (ground glass density)”, “混杂磨玻璃 (hybrid ground glass density)”, “实性 (solid)”, and etc., as the “Density” entity. Note that the descriptions like “高密度 (high density)”, “低密度 (low density)” are not annotated.

1.7 Enhancement entity

Annotating the words describing the enhancement extent of the suspected mass, e.g., “强化 (enhancement)”, “不均匀强化 (heterogeneous enhancement)”, “明显强化 (significant enhancement)”, as the “Enhancement” entity.

1.8 Shape entity

Annotating the words describing the shape of the suspected mass and its margin, e.g., “毛刺 (spiculate)”, “模糊 (blurred)”, “不规则 (irregular)”, “类圆形 (round like)”, “分叶状 (lobulated)”, and etc., as the “Shape” entity. Note that the positive descriptions, e.g., “边缘清楚 (clear margin)”, are not annotated.

1.9 Bronchus entity

Annotating the words describing the conditions of the bronchus caused by the suspected mass, e.g., “管腔狭窄 (lumen stenosis)”, “管壁增厚 (wall thickening)”, “管腔闭塞 (lumen occlusion)”, and etc., as the “Bronchus” entity. Note that we only annotate the description words as the Bronchus entity but leaving the word “支气管 (bronchus)” free in case unnecessary words are labeled into the entity in a situation where the descriptive words are far away from the position words, such as

"支气管旁见一肿物，管腔狭窄 (a lump near the bronchi with a narrowed lumen)".

1.10 Pleura entity

Annotating the words describing the conditions of the pleura caused by the suspected mass (including direct invasion and pleural nodules), e.g., "增厚凹陷 (thickening and indentation)", "关系密切 (closely related)", "分界不清 (unclear boundary)", "胸膜结节 (pleural nodules)" and etc., as the "Pleura" entity. The positive descriptions, e.g., "胸膜光滑 (smooth pleura)", are not annotated.

1.11 Vessel entity

Annotating the words or clauses describing the vessel invasion caused by the suspected mass, e.g., "包绕左肺动脉 (encircling the left pulmonary artery)", "与血管密切相关 (closely related to blood vessels)", "与右肺静脉分界不清 (unclear boundary with right pulmonary vein)", and etc., as the "Vessel" entity. Note that, unlike the "Bronchus" entities, we annotated the vessel words as the "Vessel" entity as well as the description words. Moreover, the descriptions like "远端见左肺上叶内支气管血管束增粗 (see distal thickening of bronchial vascular bundles in the left upper lobe)" not relating to great vessel invasion are not annotated.

1.12 Vertebral body entity

Annotating the clauses describing the condition of vertebral body caused by the suspected mass, e.g., "扫及椎体可见骨质破坏(scan the vertebral body and see bone destruction)", "胸 7 椎体见不规则低密度骨质破坏灶(see irregular low-density bone destruction in thoracic 7 vertebral body)", and etc., as the "Vertebral Body" Entity.

1.13 Effusion entity

Annotating the words describing the pleural effusion and pericardial effusion caused by the suspected mass, e.g., "胸水征象 (sign of pleural effusion)", "积液 (effusion)", "气液平面 (Gas-liquid plane)"and etc., as the "Effusion" entity.

1.14 Pulmonary atelectasis/obstructive pneumonitis entity

Annotating the words or clauses describing the pulmonary atelectasis or Obstructive Pneumonitis caused by the suspected mass, e.g., “肺组织不张 (atelectasis)”, “肺组织阻塞性改变 (obstructive changes in lung tissue)”, “肺不张影 (atelectasis shadow)”, “远端肺组织内见少许斑片影 (a few patchy shadows in the distal lung tissue)”, and etc., as the “Pulmonary Atelectasis/Obstructive Pneumonitis (PAOP)” entity.

2. General annotation principles

2.1 When there is a comma in the entity, e.g., “左侧支气管狭窄，管壁增厚 (left bronchi stenosis, thickened wall)”, “狭窄 (stenosis)” and “管壁增厚 (thickened wall)”, we should annotate as two entities (two “Bronchus” entities). If there is a conjunction in the entity, e.g., “边缘不规则分叶并多发毛刺 (irregular lobulated and multiple burred margins)”, “不规则分叶 (irregular lobulated)” and “多发毛刺 (multiple burred)”, it should be annotated as one entity (one “Shape” entity).

2.2 The descriptions of non-tumor-specific symptoms such as “两肺纹理增多、紊乱 (increased texture and disorder in both lungs)”, “网格样改变 (reticular changes)”, “蜂窝样改变 (honeycomb changes)”, “肺大泡 (bullae of lung)”, “肺气肿 (emphysema)”, and etc., are not annotated.
