# Supplementary material for: Automatic Extraction of Lung Cancer Staging Information From Computed Tomography Reports: Deep Learning Approach
Source: JMIR Med Inform. 2021 Jul 21;9(7):e27955. doi: 10.2196/27955 (PMC8339987; doi:10.2196/27955)
Supplement: Multimedia Appendix 3 [file medinform_v9i7e27955_app3.pdf]

Multimedia Appendix 3. The post-processing rules.

```
def _format_loc(loc):
    if not loc:
        return 0
    elif  '左'  in loc and '上' in loc:
        return '左上叶'
    elif '左'  in loc and '下' in loc:
        return  '左下叶'
    elif '右'  in loc and '上' in loc:
        return  '右上叶'
    elif '右'  in loc and '下' in loc:
        return  '右下叶'
    elif '右'  in loc and '中' in loc:
        return '右中叶'
    else:
        return loc


def parse_lymph_location(ans_dict,location_lymph,location_mass):
    if not (location_lymph and location_mass):return
    location_mass_format = _format_loc(location_mass)
    if '门' in location_lymph:
        if '双' in [location_lymph,location_mass_format] or '两' in [location_lymph,location_mass_format] or ('左肺门' not in location_lymph and '右肺门' not in location_lymph) :
            ans_dict['Q11'] = 1
            ans_dict['Q15'] = 1
        elif location_mass_format[0] in location_lymph:
            ans_dict['Q11'] = 1
        else:
            ans_dict['Q15'] = 1
    if '纵隔' in location_lymph:

        if '双' in location_mass_format  or '两' in location_mass_format  or ('L' not in location_lymph and 'R' not in location_lymph) :
            ans_dict['Q12'] = 1
            ans_dict['Q14'] = 1
        else:
            if '左' in  location_mass_format:
                mass_loc_side = 'L'
            elif  '右' in  location_mass_format :
                mass_loc_side = 'R'
```

```
        else:
            mass_loc_side = location_mass_format

        if mass_loc_side in location_lymph:
            ans_dict['Q12'] = 1
        else:
            ans_dict['Q14'] = 1
    if '隆突' in location_lymph and not ans_dict['Q13']:
        ans_dict['Q13'] = 1
    if '锁骨' in location_lymph and not ans_dict['Q16']:
        ans_dict['Q16'] = 1


def parse_mass_location(ans_dict,locations):
    main_loc = locations[0]
    main_loc_format = _format_loc(main_loc)
    for loc in locations[1:]:
        if '双'in loc or '两'in loc:
            ans_dict['Q6'] = 1
            ans_dict['Q9'] = 1
            ans_dict['Q17'] = 1
            break
        else:
            loc_num_format = _format_loc(loc)
            if loc_num_format == main_loc_format:
                ans_dict['Q6'] = 1
            else:
                if loc_num_format[0] != main_loc_format[0]:
                    ans_dict['Q17'] = 1
                else:
                    ans_dict['Q9'] = 1


def convert_triple_to_qa(filename):
    ans_dic = {
    'Q{}'.format(i):0 for i in range(1,23)
    }
    with open(filename,'r',encoding = 'utf8') as f:
        location_mass = []
        location_lymph = ''
        for line in f:
            triples = json.loads(line.strip())['triples']
            rel_lst = [tp['relation'] for tp in triples]
            for i,tp in enumerate(triples):
```

```
            if 'Negate' in rel_lst[i:min(i+2,len(rel_lst))]:
                #print('Negate')
                continue
            if 'Mass' in tp['e1']:
                if 'Location' in tp['e2'] and tp['e2_token'] not in location_mass:
#
                    location_mass.append(tp['e2_token'])
                if 'Size' in tp['e2'] and not ans_dic['Q2']:
                    ans_dic['Q2'] = 1
                elif 'Bronchus' in tp['e2'] and not ans_dic['Q3']:
                    ans_dic['Q3'] = 1
                elif 'Pleura' in tp['e2'] and '结节' not in tp['e2_token'] and not ans_dic['Q4']:
                    ans_dic['Q4'] = 1
                elif 'Pleura' in tp['e2'] and '结节' in tp['e2_token'] and not ans_dic['Q18']:
                    ans_dic['Q18'] = 1
                elif 'PulmonaryAtelectasis' in tp['e2'] and not ans_dic['Q5']:
                    ans_dic['Q5'] = 1
                elif 'Vessel' in tp['e2'] and not ans_dic['Q7']:
                    ans_dic['Q7'] = 1
                elif 'Shape' in tp['e2'] and not ans_dic['Q20']:
                    ans_dic['Q20'] = 1
                elif 'Density' in tp['e2'] and not ans_dic['Q21']:
                    ans_dic['Q21'] = 1
                elif 'Intension' in tp['e2'] and not ans_dic['Q22']:
                    ans_dic['Q22'] = 1
            elif 'Spinal' in tp['e1'] and not ans_dic['Q8']:
                ans_dic['Q8'] = 1
            elif 'Bronchus' in tp['e1'] and not ans_dic['Q3']:
                ans_dic['Q3'] = 1
            elif 'Pleura' in tp['e1'] and '结节' not in tp['e1_token'] and not ans_dic['Q4']:
                ans_dic['Q4'] = 1
            elif 'Lymph' in tp['e1'] and not ans_dic['Q10']:
                ans_dic['Q10'] = 1
            elif 'Location' in tp['e1']:
                if 'Mass' in tp['e2']:
                    ans_dic['Q1'] = 1
                    if location_mass and   ('胸' in tp['e1_token'] or '裂' in tp['e1_token'])
and not ans_dic['Q18']:
                        ans_dic['Q18'] = 1
                    location_mass.append(tp['e1_token'])

                elif 'Bronchus' in tp['e2'] and not ans_dic['Q3']:
                    ans_dic['Q3'] = 1
                elif 'Lymph' in tp['e2']:
```

```
                location_lymph = tp['e1_token']
                ans_dic['Q10'] = 1

            elif tp['e2'] in 'Effusion' and not ans_dic['Q19']:
                ans_dic['Q19'] = 1
            elif tp['e2'] in 'Pleura' and not ans_dic['Q4']:
                ans_dic['Q4'] = 1

    parse_lymph_location(ans_dic,location_lymph,location_mass[0])
    if len(location_mass) > 1:
        parse_mass_location(ans_dic,location_mass)
return [ans_dic[key] for key in ans_dic]
```
