# Supplementary material for: Automatic Extraction of Lung Cancer Staging Information From Computed Tomography Reports: Deep Learning Approach
Source: JMIR Med Inform. 2021 Jul 21;9(7):e27955. doi: 10.2196/27955 (PMC8339987; doi:10.2196/27955)
Supplement: Multimedia Appendix 4 [file medinform_v9i7e27955_app4.pdf]

Multimedia Appendix 4. Evaluation metrics.

$$\text{Precision} = \frac{\text{True Positive}}{\text{True Positive} + \text{False Positive}}$$

$$\text{Recall} = \frac{\text{True Positive}}{\text{True Positive} + \text{False Negative}}$$

$$\text{F1} = 2 \times \frac{\text{Precision} \times \text{Recall}}{\text{Precision} + \text{Recall}}$$

$$\text{Precision}_{\text{macro}} = \frac{1}{n}\sum_{i=1}^{n} \text{Precision}_i$$

$$\text{Recall}_{\text{macro}} = \frac{1}{n}\sum_{i=1}^{n} \text{Recall}_i$$

$$\text{F1}_{\text{macro}} = \frac{1}{n}\sum_{i=1}^{n} \text{F1}_i$$

$$\text{Precision}_{\text{micro}} = \frac{\sum_{i=1}^{n} \text{True Positive}_i}{\sum_{i=1}^{n} \text{True Positive}_i + \sum_{i=1}^{n} \text{False Positive}_i}$$

$$\text{Recall}_{\text{micro}} = \frac{\sum_{i=1}^{n} \text{True Positive}_i}{\sum_{i=1}^{n} \text{True Positive}_i + \sum_{i=1}^{n} \text{False Negative}_i}$$

$$\text{F1}_{\text{micro}} = 2 \times \frac{\text{Precision}_{\text{micro}} \times \text{Recall}_{\text{micro}}}{\text{Precision}_{\text{micro}} + \text{Recall}_{\text{micro}}}$$
