# Supplementary material for: Automatic Extraction of Lung Cancer Staging Information From Computed Tomography Reports: Deep Learning Approach
Source: JMIR Med Inform. 2021 Jul 21;9(7):e27955. doi: 10.2196/27955 (PMC8339987; doi:10.2196/27955)
Supplement: Multimedia Appendix 5 [file medinform_v9i7e27955_app5.pdf]

Multimedia Appendix 5. Hyper-parameters of the NER and RC models.

Multimedia Appendix 4.1 The hyper-parameters of the NER models

**ID-CNN-CRF**		**Bi-LSTM-CRF**		**BERT**	
**Hyper-parameters**	**Value**	**Hyper-parameters**	**Value**	**Hyper-parameters**	**Value**
Character embedding dimension	100	Character embedding dimension	100	Character embedding dimension	512
Optimizer	Adam	Optimizer	Adam	Optimizer	Adam
Learning rate	0.001	Learning rate	0.001	Learning rate	1e-4
Dropout	0.5	Dropout	0.5	Dropout	0.1
Gradient clip	-5 to 5	Gradient clip	-5 to 5	Layers	24
Batch size	20	Batch size	20	Batch size	8
Epoch	100	Epoch	100	Epoch	100
Number of filters	100	Hidden size	100	Hidden size	1024
Segmentation embedding dimension	20	Segmentation embedding dimension	20		
Filter width	3				
Number of layers	3				
Dilation width of each layer	1,1,2				
Block repeat times	3				

Multimedia Appendix 4.2 The hyper-parameters of the RC models

**Attention-Bi-LSTM-RSC**		**BERT-RSC**	
**Hyper-parameters**	**Value**	**Hyper-parameters**	**Value**
Character embedding dimension	100	Character embedding dimension	512
Hidden size	100	Hidden size	1024
Optimizer	Adadelta	Optimizer	AdamW
Learning rate	1.0	Learning rate	5e-4
Dropout	0.5	Dropout	0.1
$Lambda_{L2}$	1e-5	Layers	24
Batch size	20	Batch size	16
Epoch	100	Epoch	100
Decay rate	0.9		
